# Antibacterial effect of titanium oxide and cobalt-doped zinc ferrite coated stainless steel orthodontic brackets against *Streptococcus mutans* – an in-vitro study

**DOI:** 10.2340/biid.v12.44819

**Published:** 2025-10-02

**Authors:** Simarpreet Bhamra, Ritesh Singla, Suresh D. Kulkarni, Padmaja A. Shenoy, Nishu Singla, Vathsala Patil, Sandeep Kasana, Sudarshana Devadiga

**Affiliations:** a Department of Orthodontics and Dentofacial Orthopaedics, Manipal College of Dental Sciences, Manipal, Manipal Academy of Higher Education, Manipal, Karnataka, India; b Manipal Institute of Applied Physics (MIAP), Manipal Academy of Higher Education, Manipal, Karnataka, India; c Department of Microbiology, Kasturba Medical College, Manipal, Manipal Academy of Higher Education, Manipal, Karnataka, India; d Department of Public Health Dentistry, Manipal College of Dental Sciences, Manipal, Manipal Academy of Higher Education, Manipal, Karnataka, India; e Department of Oral Medicine and Radiology, Manipal College of Dental Sciences, Manipal, Manipal Academy of Higher Education, Manipal, Karnataka, India; f Resident in Orthodontics, University of Detroit Mercy, Detroit, MI, USA

**Keywords:** White spot lesions, nanoparticles, coatings, orthodontic brackets, titanium oxide, cobalt-doped zinc ferrite

## Abstract

**Background:**

White spot lesions (WSL), plaque buildup, and poor oral hygiene are all consequences of the intricate bracket patterns found in fixed orthodontic treatment. Therefore, coatings made of titanium oxide (TiO_2_) and cobalt-doped zinc ferrite (CZFO) nanoparticles were evaluated for their antibacterial qualities to address this issue.

**Objective:**

The objective of this study was to evaluate and compare the antibacterial effects of TiO_2_ and CZFO when used as surface modificants for orthodontic stainless-steel brackets in reducing the proliferation of *Streptococcus mutans (S. mutans)*.

**Materials and methods:**

The study was conducted as two main groups: a TiO_2_ group and a CZFO group. Each group was subsequently divided into three subgroups: a control group (petri dish containing *S. mutans* strain in broth without brackets), uncoated brackets (*n* = 20), and coated brackets (*n* = 20) resulting in a total of 40 brackets per group. The brackets were coated using a hydrothermal process followed by microbiological assays to determine the colony-forming units (CFU) of *S. mutans*.

**Statistical analysis:**

Results were analyzed within groups using one-way ANOVA, followed by *post hoc* Tukey tests. Differences between the two coatings were analyzed using independent Student’s t-test.

**Results:**

In their respective groups, the TiO_2_-coated and CZFO-coated brackets showed significantly lower CFUs of *S. mutans* (2.46 ± 0.15 and 2.93 ± 0.59 log_10_CFU/mL, respectively) than the control group (5.07 ± 0.24 and 4.64 ± 0.30 log_10_ CFU/mL respectively) and the uncoated brackets (4.56 ± 0.49 and 4.52 ± 0.24 log_10_ CFU/mL respectively) (Group TiO_2_-*p* < 0.001, Group CZFO-*p* = 0.004) . No significant difference in CFU was found between TiO_2_ and CZFO coatings.

**Conclusion:**

In this study, both TiO_2_ and CZFO coated brackets proved to be better than their respective control groups at reducing the viability of *S. mutans*. CZFO coated brackets exhibited antibacterial effects comparable to UV-activated TiO₂ brackets, even under visible light.

## Introduction

The oral cavity is a natural breeding ground for microorganisms like bacteria, which create organic acids, reduce pH and demineralize the surface of tooth enamel, resulting in white spot lesions (WSL), dental caries, periodontitis and other undesirable consequences [1, 2]. Materials used in orthodontic therapy tend to change the biological environment of the oral cavity, leading to a marked rise in the population of these caries-causing bacteria such as *Lactobacillus acidophilus (L. acidophilus)* and *Streptococcus mutans (S. mutans)* [3]. Numerous antibacterial treatments, including the use of antimicrobial mouthwashes and toothpastes, have been utilized therapeutically to prevent enamel demineralization. However, these traditional approaches rely heavily on full cooperation from patients, which may not always occur.

To address the above-mentioned issues, researchers have worked to include antibacterial properties into bonding adhesives and orthodontic materials such as archwires, brackets and aligners by modifying their surfaces [4]. This technology intends to alter the surface morphology, mechanical characteristics, and antibacterial qualities by creating an appropriate additional layer on the substrate’s surface.

Antibacterial coatings have been created using materials such metal oxides, metal elements, organic compounds, and others [5]. These metal nanoparticles change the permeability of microorganisms’ cell membranes and interfere with the functions of phosphorus and sulphur-containing materials, like DNA, making it tougher for them to develop resistance. Precious metal nanoparticles such as silver oxide, copper oxide, iron oxide, zinc oxide, titanium oxide (TiO_2_), etc., have garnered significant interest among different kinds of nanomaterials because of their superior optical, electrical, and catalytic capabilities [6].

TiO_2_ is a photosensitive material that produces reactive oxygen species (ROS) and hydroxyl (OH) radicals when exposed to ultraviolet (UV) radiation which are extremely reactive when they encounter organic substances [7]. This idea has led to increased interest in TiO_2_’s antimicrobial qualities. It can be found in crystal forms of rutile, anatase, and brookite. Anodic oxidation produces an anatase phase of TiO_2_, whereas thermal oxidation produces a rutile phase. These coatings also exhibit antibacterial and antiadhesive qualities against *L. acidophilus, Candida albicans (C. albicans)*, and *S. mutans* [8]. As effective as TiO_2_ is, concern has been raised about its cytotoxic effects, particularly in the rutile phase. Hence, the anatase phase is preferred over rutile phase. Despite its increased cost, the passivating coating of TiO_2_ is helpful in mitigating the allergic qualities of nickel while maintaining its relevance.

On the other hand, owing to its high permeability, high magnetization, high electrical resistivity, and low cost, nanosized spinel zinc ferrite (ZnFe_2_O_4_) powders have attracted a lot of attention for their diverse technological applications [9]. One of the most researched visible light photocatalysts, ZnFe_2_O_4_ exhibits effective absorption of visible light because of its low bandgap (1.9 eV), good stability for reuse, and magnetic separability [10]. However, owing to ZnFe_2_O_4_’s fast recombination, photogenerated electron-hole pairs last less time than the time needed to carry out reduction/oxidation reactions at the valence and conduction bands, respectively. To curb this issue, Co^2+^ substituted ZnFe_2_O_4_ (cobalt-doped zinc ferrite [CZFO]) was synthesized which reduced the bandgap, allowing a wide range of light absorption ranging from visible to near infrared (NIR) light as well as increased the life span of ROS thus produced. As there is no evidence in literature regarding CZFO coatings on brackets, this material has been chosen as the second coating material in our study.

Thus, the purpose of this research was to evaluate and compare the antibacterial effects of TiO_2_ and CZFO when used as surface modificants for orthodontic stainless-steel brackets. The null hypothesis tested was that there would be no significant difference in the antibacterial effects of TiO₂ and CZFO coatings when used as surface modifications on orthodontic stainless-steel brackets against *S. mutans*.

## Materials and methods

Approval for this study was obtained from Institutional Ethics Committee (IEC2: – 200/2023). Sample size was calculated considering significance level (α) of 0.05 and power of study (β) to be 80%. σ is the assumed standard deviation of 0.3 (assumed to be equal for all the groups), and the minimum expected difference between the two means is 0.43 (taken from a previous study). Assuming possible losses of 30%, the number of subjects per group was adjusted to be around 20 samples per group [11]. Subsequently, 80 samples of stainless steel MBT (McLaughlin, Bennett, Trevisi) premolar brackets (0.022” × 0.028” slot size) were procured from American Orthodontics following which, two main groups were formed:

GROUP TiO_2_ and GROUP CZFO each with three subgroups (Table 1).

**Table 1:** Presents the two experimental groups along with their respective three subgroups.

Group TiO_2_	Group CZFO
**CONTROL**- petri dish containing *S.mutans* strain in broth without brackets	**CONTROL-** petri dish containing *S.mutans* strain in broth without brackets
**UNCOATED**- contained 20 uncoated brackets	**UNCOATED-** contained 20 uncoated brackets
**COATED**- contained 20 TiO_2_ coated brackets	**COATED-** contained 20 CZFO coated brackets

CZFO, cobalt doped zinc ferrite.

## Coating procedure

Coating on brackets was done in the Department of Atomic and Molecular Physics, Manipal Academy of Higher Education (MAHE) using the hydrothermal method which is a type of solution-based method wherein brackets were dipped in their respective solutions following which heat was applied to the substrates.

### TiO_2_ deposition on stainless steel brackets

**Figure 1:**
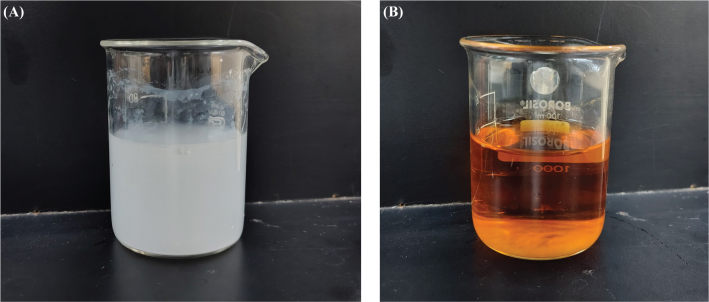
(A) TiO2 solution used for coating. (B) Cobalt doped zinc ferrite solution used for coating.

600 µL (2 mmol) of titanium tetra isopropoxide was added to 30 mL of isopropyl alcohol while stirring. Then, 2.25 mL of deionized water was added dropwise, forming a white precipitate, Ti[OCH(CH_2_)_2_]_4_. HNO_3_ was added to adjust the pH to 5, and stirring continued for 30 min. The mixture (Figure 1A) was transferred to an autoclave along with 20 cleaned stainless steel brackets and heated at 200°C for 2 h. After cooling, the brackets were removed, rinsed with water, and dried in a hot air oven [12].

### Cobalt-doped zinc ferrite deposition on stainless steel brackets

161.6 mg (0.4 mmol) of Fe(NO_3_)_3_•9H_2_O was dissolved in 10 mL of ethylene glycol (EG) to form a clear solution. Then, 0.022 mmol of Co(NO_3_)_2_•6H2O and 0.18 mmol of Zn(NO_3_)_2_•6H2O were added. A transparent solution of 1 g sodium acetate tri-hydrate in 10 mL of EG was mixed in, followed by 20 min of vigorous stirring. The mixture (Figure 1B) was transferred to an autoclave with 20 cleaned stainless steel brackets and heated at 200°C for 8 h. After heating, the autoclave was allowed to cool naturally, and the brackets were removed, rinsed with water, and dried in a hot air oven [13].

**Figure 2:**
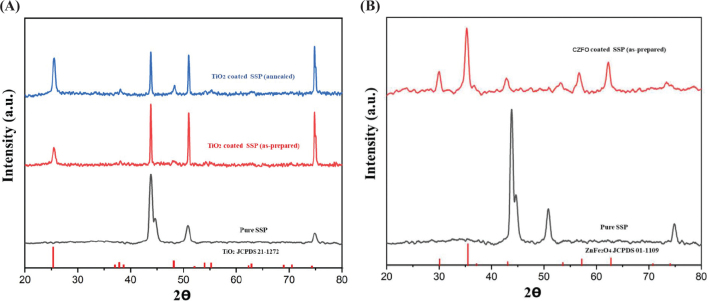
(A) X-ray diffractometry patterns of TiO2 coated stainless steel plate. (B) X-ray diffractometry patterns of Cobalt doped zinc ferrite coated stainless steel plate.

**Figure 3:**
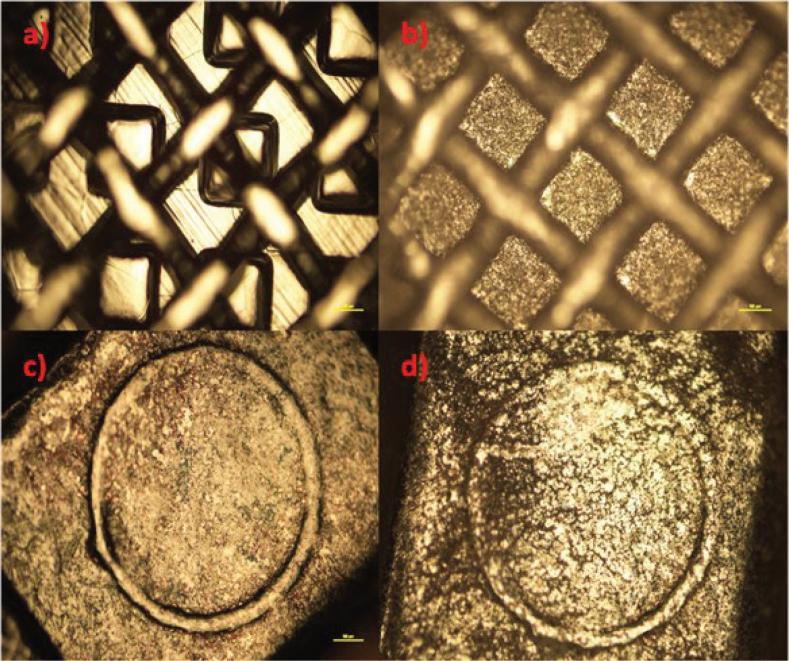
(A) and (C) Microscopic images of uncoated brackets, (B and D) images of TiO_2_ coated brackets.

**Figure 4:**
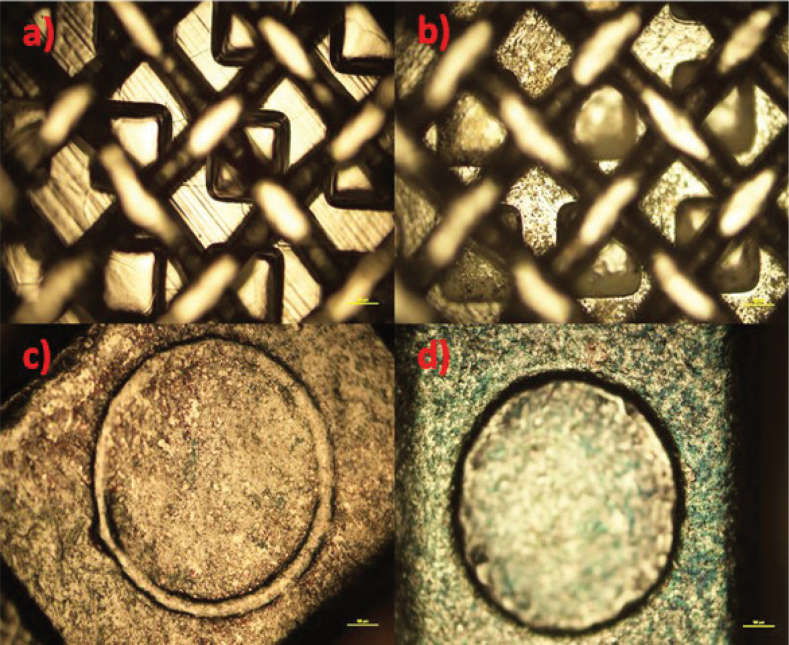
(A) and (C) Microscopic images of uncoated brackets, (B and D) images of cobalt doped zinc ferrite coated brackets.

**Figure 5:**
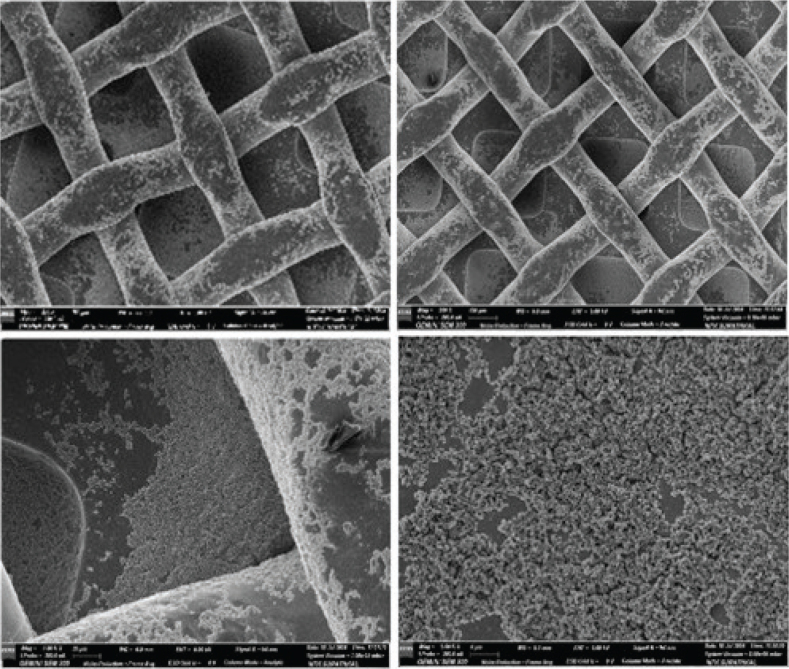
Field Emission Scanning Electron Microscopy images showing TiO_2_ coating on stainless steel brackets.

**Figure 6:**
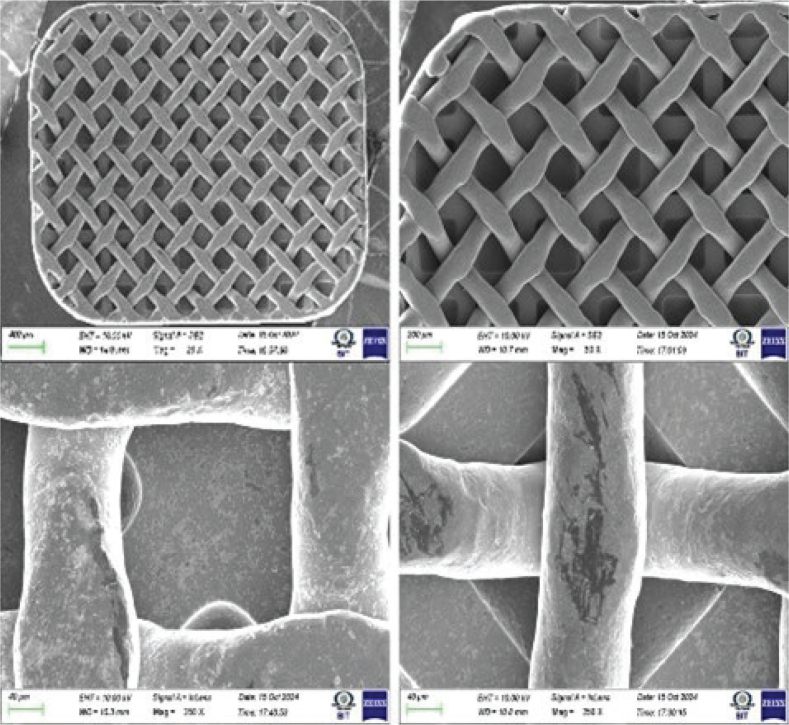
Field Emission Scanning Electron Microscopy image showing cobalt doped zinc ferrite coating on stainless steel brackets.

TiO_2_ (anatase) and CZFO deposition on stainless steel plates was confirmed by X-ray diffractometry (XRD) characterization (Figure 2A and B respectively), whereas on brackets, it was confirmed by optical microscope images of coated and uncoated brackets (Figures 3 and 4 respectively). Subsequently, their thickness and uniformity were confirmed using field emission scanning electron microscopy (FESEM) images (Figures 5 and 6 respectively).

TiO_2_ particles were about 300 nm in size and nearly spherical, with a uniform and homogenous coating of approximately 1.2 μm. Similarly, the CZFO coating was uniform and homogenous, approximately 450 nm thick, with particle sizes of 30 to 40 nm, also nearly spherical.

## Microbiological assessment

After nanoparticle coating, the brackets were characterized and disinfected to prepare them for the next part of the study, which was the antibacterial assessment of the coated brackets against the control. For disinfecting brackets, 40 uncoated, 20 TiO_2_ coated, and 20 CZFO coated brackets were immersed in an ultrasonic cleaner for 5 mins after which the brackets were dried, sealed in separate packets and autoclaved to ensure disinfection.

As the reservoir for our investigation, 5 mL of brain heart infusion (BHI) broth was inoculated with *S. mutans* and subsequently incubated for 24 h at 37°C. A clinical strain of *S. mutans* was used in the investigation isolated from patients with periodontal infection and dental caries. Following the 24-h incubation period, the reservoir was diluted using BHI broth to reach an optical density of 4.0 which was confirmed using the DensiCHEK by BioMerieux, France (microbiological equipment for measuring turbidity standard).

1 mL of this stock culture was diluted in 100 mL of sterile normal saline, following which 10 mL of this diluted solution was added into the following petri dishes.

Group TiO_2_ Petri dishes (control, uncoated and coated brackets) were exposed to UV-A black light for 60 min within the Biosafety Cabinet Class II, whereas petri dishes in Group CZFO (control, uncoated and coated brackets) were exposed to halogen light for the same amount of time. Following exposure, BHI agar plates were serially inoculated with a 100 μL dilution of the bacterial culture, ranging in dilutions from 10^−1^ to 10^−4^, and the plates were incubated for 48 h at 37°C after which colonies formed were recorded using manual plate count method.

### Statistical analysis

The statistical software program SPSS 29.0 (SPSS Inc., Chicago, IL) was used to analyze the study data. To evaluate the mean and standard deviation of each group, descriptive statistics were used. The Shapiro-Wilk test was used to determine the normality of the data, and the results showed a normal distribution (*p* > 0.05), allowing for parametric testing. Within each of the two main groups, ‘One-way ANOVA test’ and ‘Tukeys *post hoc* test’ were used to test for differences between the three subgroups. The Independent Students’ *t*-test was utilized to compare the effect of the CZFO and TiO_2_ coatings. The significance level was set at *p* < 0.05.

Colony-forming units (CFU) have been expressed in logarithmic form (log₁₀ CFU/mL) to simplify the interpretation of bacterial counts that often span a wide range – from hundreds to millions – by converting them into manageable numbers. This transformation not only makes data easier to compare but also helps normalize skewed distributions, allowing for a more accurate statistical analysis using parametric tests. Additionally, microbial growth and reduction typically follow a logarithmic pattern, so expressing CFUs in this way reflects the biological behavior of bacteria and provides clearer visualization of changes across experimental groups.

## Results

The study evaluated *S. mutans* colony formation in different groups in terms of log 10 CFU/mL, and the results are presented in Table 2. In the TiO_2_ group, the control group had the highest count, followed by the uncoated brackets, while the coated brackets showed the lowest count. Similarly, in the CZFO group, the control group had the highest count, followed by the uncoated brackets, and the coated brackets.

**Table 2:** Intragroup comparison of *S. mutans* colonization among TiO₂ and CZFO subgroups (Log₁₀ CFU/mL).

Groups	Subgroups	Log 10 CFU/mL (mean ± SD)	‘*p*’ value
GROUP TiO_2_	Control	5.07 ± 0.24	**< 0.001**
	Uncoated	4.56 ± 0.49	
	Coated	2.46 ± 0.15	
GROUP CZFO	Control	4.64 ± 0.30	**0.004**
	Uncoated	4.52 ± 0.24	
	Coated	2.93 ± 0.59	

CZFO: cobalt doped zinc ferrite; FFU: colony-forming units.

One-way ANOVA Test, ‘*P*’ value ≤ 0.05 is considered statistically significant, SD- Standard deviation.

Within each of the two groups (TiO_2_ and CZFO), the one-way ANOVA found significant differences between the three subgroups. Subsequently, the post-hoc tests found both coatings to have significantly lower CFU than their respective control group and uncoated group, while no differences were found between the control and uncoated groups (Table 3).

**Table 3:** Pairwise comparison of antibacterial effects between control, uncoated, and coated brackets within TiO₂ and CZFO Groups (Log₁₀ CFU/mL).

Groups	Pair-wise group comparison	Mean difference log10 CFU/mL	*p*
GROUP TiO_2_	Control versus uncoated	0.51	0.213
	Control versus coated	2.61	**< 0.001**
	Uncoated versus coated	2.10	**0.001**
GROUP CZFO	Control versus Uncoated	0.12	0.928
	Control versus coated	1.72	**0.005**
	Uncoated versus coated	1.59	**0.007**

*CZFO*, cobalt doped zinc ferrite; CFU: colony-forming units.

Post hoc Tukey Test, *p* ≤ 0.05 is considered statistically significant.

Finally, there was no significant difference between the two coatings (Table 4).

**Table 4:** Comparison of antibacterial efficacy between CZFO and TiO₂-coated brackets against *S. mutans* (Log₁₀ CFU/mL).

Bacterial count	Group – CZFO (mean ± SD)	Group- TiO_2_ (mean ± SD)	Mean difference	*p*
Log 10 CFU/mL	2.93 ± 0.59	2.46 ± 0.15	0.47	0.317

CZFO, cobalt doped zinc ferrite; CFU: colony-forming units.

Independent students’ *t*-test, *p* ≤ 0.05 is considered statistically significant, SD- Standard deviation.

**Graph 1:**
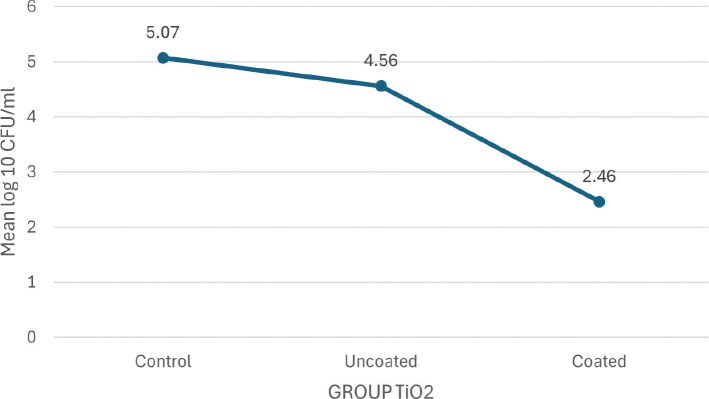
Mean plot of Log₁₀ CFU/mL units of *S. mutans* between subgroups of Group TiO_2_.

**Graph 2:**
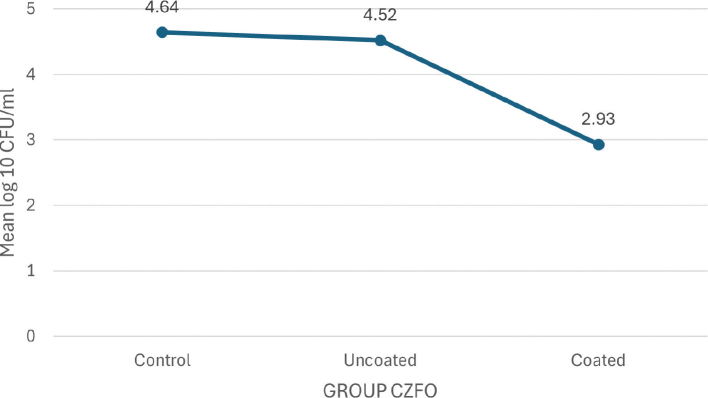
Mean plot of Log₁₀ CFU/mL units of *S.mutans* between subgroups of Group cobalt doped zinc ferrite.

The mean plot of log_10_ CFU/mL of *S. mutans* between the subgroups of group TiO_2_ (Graph 1) and group CZFO (Graph 2) shows a negatively skewing graph towards their coated subgroups respectively, thereby confirming that lesser number of colonies were seen in these subgroups as compared to those seen in control and uncoated groups.

## Discussion

The biological milieu that the biomaterial brackets create is crucial for the early adherence of bacteria that form biofilms, which leads to the failure of orthodontic treatments [14]. A study conducted by Ahn et al. in 2002 showed how stainless-steel brackets provide the ideal binding surface for the biofilm-forming *S. mutans* [15]. Accumulation of bacteria and plaque surrounding the orthodontic appliances may be the pathologic process leading to WSL. Studies have shown that adding nanoparticles of Ag (silver), Au (gold), ZrO_2_ (zirconium oxide), and TiO_2_ to orthodontic adhesives can enhance the mechanical properties of materials, such as compressive, tensile, and shear bond strength. Moreover, when applied to the orthodontic bracket, it will produce an antibacterial film effect, which can effectively reduce the rate of dental caries in orthodontic patients [16]. Lately, this has drawn attention and served as a topic of our study. Therefore, in an effort to resolve this problem, we have investigated the antibacterial qualities of coatings (TiO_2_ and CZFO) on stainless steel orthodontic brackets.

The application of photocatalytic TiO_2_ has garnered increased interest as one pertinent topic in this regard. Due to its intrinsic fundamental properties, TiO_2_ has emerged as a superior photocatalytic material for a wide range of industrial and biomedical applications. With its antibacterial properties, titanium can be employed as a nanomaterial with a wide range of potential applications in limiting bacterial growth and consequently infections caused by these microorganisms [17]. Hence, it was chosen as one of the two materials used in our study for coating brackets.

Magnetic nanoparticles (MNP), especially iron oxide nanoparticles like spinel ferrites, are gaining attention for biomedical applications due to their unique magnetic properties, chemical durability, biological compatibility, and affordability, making them suitable for innovative medical technologies [18]. Among these spinels, ZnFe_2_O_4_ nanoparticles have acquired interest in a range of clinical uses because of their biocompatible nature, reduced toxicity in comparison to other metal ferrites, chemical resistance, facile and repeatable synthesis, low saturation magnetization, and photo-induced catalytic reactions [19]. ZnFe_2_O_4_ has a limited lifespan for photogenerated electron-hole pairs, hindering essential reduction and oxidation reactions. Doping it with Co^2+^ to create CZFO effectively addressed this issue by lowering the bandgap and expanding the light absorption spectrum to include visible and NIR light. As a result, CZFO nanoparticles were chosen for coating brackets due to their enhanced performance in relevant applications.

The reduced number of *S. mutans* colonies seen in TiO_2_ coated group as compared to the control group and the uncoated brackets group is in accordance with previous studies [14, 20–22], where it was observed that TiO_2_ coatings on brackets reduced the viability of *S. mutans* colonies. The reason behind this could be the production of ROS being released upon subjecting the above-mentioned nanoparticles to UV light, specially UV-A. This antibacterial effect of TiO_2_ coated brackets and wire surfaces after subjecting them to UV light is known to occur due to ‘photocatalysis’. TiO_2_ being a semiconductor material has a high energy band gap; therefore, when exposed to UV radiation with energy above the band gap, they produce ROS, which confers antibacterial action [23]. This has shown promise in combating cariogenic bacteria such as *L. acidophilus* and *S. mutans* [24]. TiO_2_ experiences photoactivation in the presence of UV light, which produces electron-hole pairs. This activation results in the production of superoxide anions and OH radicals, which take part in a sequence of oxidation and redox reactions to break down the organic molecules [25]. Oxidation makes cells more permeable and permits unrestricted intracellular fluid outflow which changes the permeability of the bacteria to ROS, resulting in lipid peroxidation, thereby altering the cell membrane’s structure and function and ultimately causing cell death [26].

TiO₂ coatings reduce surface roughness, leading to decreased bacterial adhesion, less plaque formation, and fewer periodontal diseases and WSL, aligning with previous research findings [20, 21].

The crystalline and amorphous substances that make up TiO_2_ nanoparticles namely, anatase, rutile, and brookite were classified based on physical characteristics like surface area and optical band gap [27]. While rutile was found to be more stable under thermodynamic conditions, anatase structures were seen to be photoactive [24] and less cytotoxic as compared to rutile phase structures [25]. Therefore, for our study, we chose the anatase phase of TiO_2_ to be used as a nanoparticle coating on brackets.

Another key finding of our study was the reduced S. mutans colonies seen in the petri dish containing brackets coated with CZFO as compared to the control groups and the uncoated bracket group. The effective photocatalytic property of CZFO nanoparticles is the result of its low energy band gap and the presence of ferric ions (Fe^3+^). As per the Fenton equations, the ferric ions combine with the ROS to form free OH radicals, which in turn generate hydrogen peroxide, which destroys the bacteria [28]. Another advantage of using Co^2+^ as a dopant is the increased quantity of ROS produced. Its optical and magnetic characteristics are extremely remarkable, and its strong reactivity is caused partly because of its short particle size contributing to a large surface area for bacterial interactions. This narrow energy gap of CZFO particles allows it to undergo photocatalysis in the presence of visible light. The fact that the CZFO coatings are activated by visible light while the TiO_2_ coatings require UV light, was the reason for choosing to include CZFO coating in this study. Visible blue light is preferable as it effectively inactivates pathogens and degrades contaminants, making it a promising microbicidal tool for clinical and public health use, while ensuring safety for host cells. This contrasts with UV technologies, which can harm the host environment [29].

A further extensively proposed mechanism involves the antibiotic’s ‘self-promoted uptake’ through the lipopolysaccaride surface of the bacteria’s outer membrane. This implies that the nanoparticles’ interaction with the charged outer membrane causes channel development in the cytoplasmic membrane which ultimately leads to cell death through a ‘Carpet’ or ‘Barrel-Stave’ process. A study done by Haghniaz et al. shows that CZFO nanoparticles have bactericidal effectiveness comparable to that of tetracycline [19]. They also concluded that, a Gram-positive bacterial species, *S. aureus* was more sensitive to ZnFe_2_O_4_ nanoparticles rather than the Gram-negative bacteria *E. coli*.

Despite superior photocatalytic properties of TiO₂ under UV-A light, facilitating the generation of ROS, this study found no statistically significant difference in the reduction of *S. mutans* CFU between the TiO₂ coated brackets and the CZFO coated brackets. This suggests that both TiO₂ and CZFO coatings produced comparable levels of ROS, contributing significantly to the antibacterial activity observed in both coated groups.

Most dental materials that are utilized in the oral cavity run the risk of causing allergic reactions or affecting the nearby tissues. Though TiO_2_ has been regarded as a biocompatible material, as a result of mechanical stress and wear, the TiO_2_ layer degrades and releases corrosion byproducts from the bracket surface, which can lead to diseases and be hazardous [30]. These byproducts of corrosion have the potential to react in osteogenic cells, fibrotic tissue, and blood. Toxic MNPs, on the other hand, have the potential to negatively impact metabolic activity, cell viability, and proliferation rate, as well as distort the treatment’s therapeutic effectiveness. The properties, dosage, and uses of MNPs, especially iron oxide, are the primary determinants of their toxicity to living organisms [31]. Build-up of iron oxide nanoparticles can cause apoptosis as it changes the functioning of macrophages. It has also been seen that CZFO nanoparticles can be toxic to liver and kidney tissues as evidenced by increased levels of AST, ALT, urea and creatinine [32]. Therefore, prior to being applied to orthodontic materials, it is necessary to ascertain the maximum lethal dose of TiO_2_ and CZFO nanoparticles.

## Conclusion

This study on antibacterial effects of TiO_2_ and cobalt-doped zinc ferrite coated stainless steel orthodontic brackets offers valuable insights, and the following conclusions were drawn.

TiO_2_ coated brackets are better than uncoated brackets for controlling the growth of *S. mutans* colonies.

Similarly, CZFO coated brackets are better than uncoated brackets.

CZFO coated brackets showed similar antibacterial effect as TiO_2_ coated brackets exposed to UV light, even under visible light conditions.

## Limitations of the study

The *in vitro* investigation has limitations for real intraoral applications, including untested cytotoxicity and stability of CZFO and TiO_2_ coatings, as well as unknown interactions with saliva. The requirement to expose each bracket to visible or UV light for 60 min complicates practical application due to UV exposure risks and ensuring adequate light coverage on all surfaces. Additionally, focusing solely on *S. mutans* restricts the relevance to other oral pathogens, raising concerns about their applicability to diverse oral microbiota. These significant issues must be addressed before considering clinical application.

## Future scope of research

Further investigations are needed to evaluate the intraoral stability and cytotoxicity of the coatings. Future research should include a wider range of oral bacteria and assess factors affecting antibacterial efficacy, such as light intensity, wavelength, and distance from the light source, as these are crucial for the coatings’ effectiveness against bacteria.

## Data Availability

The study data set is available on request from xxx.
